# Cognitive and Orofacial Intersections: prevalence of temporomandibular disorders and bruxism in individuals with symptoms of attention deficit hyperactivity disorder

**DOI:** 10.1590/1678-7765-2025-0771

**Published:** 2026-03-23

**Authors:** Arthur Alexis Valentin Caron, Rodrigo Lorenzi Poluha, Marlon Ferreira Dias, Dyanne Medina Flores, Alfonso Sanchéz-Ayala, Justin Durham, Giancarlo De la Torre Canales

**Affiliations:** 1 Egas Moniz School of Health and Science Egas Moniz Center for Interdisciplinary Research Almada Portugal Egas Moniz School of Health and Science, Egas Moniz Center for Interdisciplinary Research (CiiEM), Caparica, Almada, Portugal.; 2 Universidade Estadual de Maringá Departamento de Odontologia Paraná Brasil Universidade Estadual de Maringá, Departamento de Odontologia, Paraná, Brasil.; 3 Universidade Estadual Paulista Faculdade de Odontologia de Araraquara Departamento de Materiais Odontológicos e Prótese Araraquara Brasil Universidade Estadual Paulista - UNESP/FOAr, Faculdade de Odontologia de Araraquara, Departamento de Materiais Odontológicos e Prótese, Araraquara, Brasil.; 4 Universidade de São Paulo Faculdade de Odontologia de Bauru Departamento de Prótese Bauru Brasil Universidade de São Paulo - USP/FOB, Faculdade de Odontologia de Bauru, Departamento de Prótese, Bauru, Brasil.; 5 Universidade de Ponta Grossa Departamento de Odontologia Ponta Grossa Brasil Universidade de Ponta Grossa, Departamento de Odontologia, Ponta Grossa, Brasil.; 6 Newcastle University School of Dental Sciences Newcastle-Upon-Tyne United Kingdom Newcastle University, School of Dental Sciences, Newcastle-Upon-Tyne, United Kingdom.; 7 Karolinska Institutet Department of Dental Medicine Division of Oral Rehabilitation Huddinge Sweden Karolinska Institutet, Department of Dental Medicine, Division of Oral Rehabilitation, Huddinge, Sweden.

**Keywords:** Attention deficit disorder with hyperactivity, Temporomandibular disorders, Bruxism

## Abstract

Objective. Despite emerging evidence pointing to a comorbidity between temporomandibular disorders (TMD), bruxism, and neuropsychiatric conditions such as Attention-Deficit/Hyperactivity Disorder (ADHD), limited studies have explored this relationship in adult populations. This study aimed to investigate the prevalence of painful TMD and self-reported bruxism in adults with highly consistent ADHD symptoms. Methodology. In total, 90 adult participants were divided into two groups based on the Adult ADHD Self-Report Scale Screener, G1: individuals with highly consistent ADHD symptomatology (n=60), and G2: controls (n=30). All participants completed validated instruments assessing TMD (TMD Pain Screener), self-reported bruxism (OBC), anxiety (GAD-7), somatization (PHQ-15), perceived stress (PSS), sleep quality (PSQI), pain hypervigilance (PVAQ), and jaw function (JFLS-8). The chi-square test, adjusted using Bonferroni criteria for multiple comparisons, and the Mann-Whitney U-test were used. Results. A higher prevalence of women (p=0.006), South Americans (p=0.008), and individuals with low/lower middle income (p=0.0001) was found in G1. G1 showed a significantly higher prevalence of painful TMD (p=0.001) and awake bruxism (p=0.013) compared to G2. Participants in G1 also exhibited significantly higher levels of anxiety, somatization, stress, poor sleep quality, pain hypervigilance, and jaw limitation (p<0.05). No significant differences in sleep bruxism were found between groups (p>0.05). Conclusion. These findings suggest a potential behavioral and emotional vulnerability to orofacial disorders in adults with highly consistent ADHD symptoms.

## Graphical abstract

**Figure:**
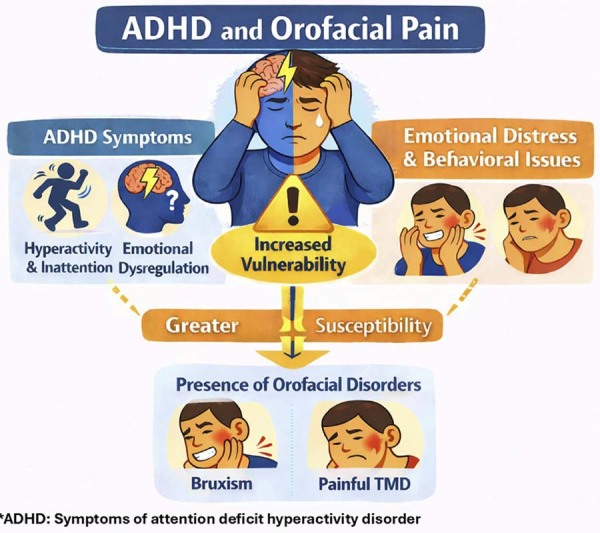
This article is derived from a master's dissertation available at http://hdl.handle.net/10400.26/61029

## Introduction

Temporomandibular Disorders (TMD) are musculoskeletal conditions that affect the temporomandibular joints (TMJ), masticatory muscles, and associated structures.^1^Affecting approximately 34% of the population,^2^ TMD may present as orofacial pain, limited mouth opening, joint sounds, and stiffness.^3^ Psychosocial factors play a critical role in exacerbating pain and impairing quality of life,^4^ and their association with TMD is well established.^5^ Conditions such as anxiety, stress, behavioral impulsivity, and neuropsychiatric disorders—particularly Attention-Deficit/Hyperactivity Disorder (ADHD)—may influence pain and, if present in individuals living with TMD, could theoretically contribute to its persistence or exacerbation.^6,7^

ADHD is characterized by a persistent pattern of inattention, hyperactivity-impulsivity, or both, typically manifesting in early or middle childhood.^8^The prevalence of ADHD in adults ranges from 2% to 4.4%, with symptoms that interfere with academic, occupational, or social functioning.^8^ Previous studies indicate high heritability, with most estimates ranging between 70% and 80%.^9^ Its etiology encompasses genetic, environmental, prenatal, and perinatal factors.^10^ Although psychostimulant medications are widely recommended for ADHD management, most medical organizations advocate beginning treatment with psychoeducation and behavioral strategies, particularly in cases of mild symptoms and functional impairment.^11^

Research has demonstrated that adults exhibiting ADHD symptoms experience significantly higher levels of pain and more severe muscular and articular problems.^7^ ADHD is a frequent and disabling condition^12^ associated with personality, mood, and anxiety disorders,^13^ which may influence orofacial pain conditions such as TMD^14^and bruxism (a repetitive jaw-muscle activity characterized by clenching or grinding of the teeth and/or by bracing or thrusting of the mandible).^15^ A recent study found that patients diagnosed with ADHD and taking stimulant medications may be more likely to report clenching, jaw pain, and headache than those without an ADHD diagnosis and not taking stimulants.^16^

Despite emerging evidence, the relationship between ADHD, TMD, and bruxism remains scarce and inconclusive.^7,17,18^ Moreover, previous studies have focused primarily on children^17^and have not examined bruxism in relation to both TMD and ADHD.^7,18^ To clarify these associations in adults, the present study aimed to evaluate the prevalence of painful TMD and self-reported bruxism in a convenience sample of adults with symptoms highly consistent with ADHD, providing an exploratory perspective for future studies.

## Methodology

This exploratory cross-sectional online study received approval from the Research Ethics Committee of Egas Moniz School of Health and Sciences (PT-229/24) and adhered to the Declaration of Helsinki. Before the initiation of this study, all volunteers were informed about its objectives and methodology and were asked to sign an informed consent form. Data reporting followed the Strengthening the Reporting of Observational Studies in Epidemiology (STROBE) guidelines.^19^

### Participants

The sample consisted of individuals with or without symptoms consistent with ADHD, according to the validated European Portuguese version of the Adult ADHD Self-Report Scale (ASRS) Screener.^20^ This is a six-question, self-administered instrument that uses a 5-point Likert scale to assess ADHD symptomatology (0=Never, 1=Rarely, 2=Sometimes, 3=Often and 4=Very Often). The ASRS has been shown useful in identifying adult ADHD symptomatology in the general population in the United States (US) and other countries. It has a positive predictive value of 0.94, a negative predictive value of 0.24, sensitivity of 68.7%, specificity of 99.5%, and a total classification accuracy of 97.9%.^20-22^

Inclusion criteria comprised individuals of both sexes, aged from 18 to 50 years, with or without painful TMD, according to the TMD Screener included in the Diagnostic Criteria for Temporomandibular Disorders (DC/TMD),^23^ and with or without self-reported bruxism, based on items #1 (sleep bruxism) and #3, #4, #5, and #6 (awake bruxism) of the Oral Behavior Checklist (test-retest reliability ranges from 0.6 to 0.98).^24^ Exclusion criteria included individuals aged under 18 years, those undergoing orthodontic treatment, and those who self-reported other diagnosed psychological disorders in addition to ADHD in an open question included in the online questionnaire.

This investigation was designed as an exploratory study and was not statistically powered, as insufficient prior data were available to support a formal sample size estimation. Accordingly, the study aimed to recruit at least 24 participants per group, as suggested by a previous cross-sectional study,^7^ to obtain an indication of the variability of the parameters of interest and inform future studies.

### Study protocol

Participants were invited to complete a structured online questionnaire, developed using *Google Forms.* The survey included an informed consent form, which participants signed prior to participation. The questionnaire consisted of three parts: (a) sociodemographic and occupational data; (b) assessment of symptoms highly related with ADHD through the ASRS-v1.1; and (c) evaluation of TMD through the TMD Pain Screener included in the DC/TMD, as well as psychosocial distress assessed through Axis II instruments and other questionnaires also included in the DC/TMD. Study advertisement and recruitment were performed via websites and social media platforms specific to the ADHD community and included a link to the questionnaire. Data were collected from November 2024 to April 2025. The sample was then divided into two groups: G1 (n=60), volunteers with symptoms highly consistent with ADHD; and G2 (n=30), volunteers without symptoms highly consistent with ADHD. Group assignment was based on reported ADHD symptoms; no formal ADHD diagnosis was performed.

### Assessment instruments

The European Portuguese versions of the following questionnaires were used:

#### Jaw functional limitation

The Jaw Functional Limitation Scale (JFLS-8) was used to evaluate functional limitations of the masticatory system in general. In addition to masticatory limitation, the instrument assesses limitation of vertical mobility and limitation of emotional and verbal expression. It consists of eight items, each rated on a numerical scale from 0 to 10 (0 indicates no limitation; 10 indicates severe limitation).^25^To the best of our knowledge, no validated cut-off point has been established; however, higher JFLS-8 scores reflect progressively greater jaw functional limitation.

#### Anxiety symptoms

Anxiety symptoms were assessed using the Generalized Anxiety Disorder (GAD-7) scale. This self-administered questionnaire consists of seven items in which participants report the degree to which they experience each described thought or feeling. The items include: (1) feeling nervous, anxious, or on edge; (2) not being able to stop or control worrying; (3) worrying too much about different things; (4) trouble relaxing; (5) being so restless that it is hard to sit still; (6) becoming easily annoyed or irritable; and (7) feeling afraid as if something awful might happen. Response options were “not at all,” “several days,” “more than half the days,” and “nearly every day,” scored as 0, 1, 2, and 3, respectively. Anxiety was classified as minimal (0-4), mild (5-9), moderate (10-14), and severe (15-21). A score above 8 indicates clinically significant anxiety symptoms.^26^

#### Somatization symptoms

Physical symptoms were assessed using the Patient Health Questionnaire (PHQ-15). This brief, self-administered instrument is used to detect somatization and monitor the severity of somatic symptoms. It comprises 15 items assessing common somatic symptoms, including back pain, joint pain, abdominal discomfort, headaches, fatigue, and sleep disturbances, over the preceding four weeks. Each item is scored from 0 (“not bothered at all”) to 2 (“bothered a little” or “bothered a lot”), yielding a total score ranging from 0 to 30. Higher scores indicate greater somatic symptom severity, with categories including minimal (0–4), low (5–9), medium (10–14), and high (15–30) levels of somatic symptom severity. ^27^

#### Perceived Stress

The Perceived Stress Scale (PSS) was applied to assess the extent to which participants appraised situations in their lives as stressful and the degree to which they perceived themselves as able to cope with these situations. Participants indicated how often they had thought or felt this way using a 5-point scale (never [0], almost never [1], sometimes [2], often [3], and very often [4]). The PSS yields a single overall perceived stress score obtained by summing the item scores. Total scores ranging from 0–13 indicate low stress, 14–26 moderate stress, and 27–40 high perceived stress.^28^

#### Sleep quality

Sleep quality over the past month was assessed using the Pittsburgh Sleep Quality Index (PSQI). This self-administered questionnaire comprises 19 items grouped into seven components, each scored from 0 to 3. The components assess subjective sleep quality, sleep latency, sleep duration, habitual sleep efficiency, sleep disturbances, use of sleep medication, and daytime dysfunction, distinguishing individuals who sleep “well” or “poorly” and providing clinical information about different sleep disorders. The component scores are summed to generate a global score, which ranges from 0 to 21, with higher scores indicating poorer sleep quality. Global scores >5 are generally used to indicate poor sleep.^29,30^

#### Vigilance related to pain

The Pain Vigilance and Awareness Questionnaire (PVAQ) was used to assess attentional processes, conscious awareness, and vigilance related to pain. Participants indicated how frequently each statement had been true for them during the past two weeks. The questionnaire consists of 16 items rated on a 6-point scale (0=never to 5=always). Total scores are obtained by summing item responses, with higher scores indicating greater vigilance and awareness of pain.^31^

#### Oral behaviors checklist (OBC)

The OBC is a self-report instrument that assesses a range of jaw-related behaviors. It consists of 21 items referring to behaviors experienced in the past 30 days. Each item is rated on a scale from 0 (“none of the time”) to 4 (“all of the time”). Total scores are classified as normal (0–16), moderate (17–24), or severe (25–62) oral behaviors.^24^

Self-reported sleep and awake bruxism were identified using the corresponding item from the OBC. Participants were classified as positive for self-reported sleep bruxism (SB) if they responded “yes” with a reported frequency of at least “1–3 nights per week” to the question: *“Do you grind or clench your teeth during sleep based on any information you may have?”* Participants were classified as positive for self-reported awake bruxism (AB) if they responded “yes” with a reported frequency of at least “some of the time” to any of the following items: “*Grind teeth together during waking hours?*”*,* “*Clench teeth together during waking hours?*”*,* “*Press, touch, or hold teeth together other than while eating?*”, and “*Hold, tighten, or tense muscles without clenching or bringing teeth together?*”^32^

## Statistical analysis

Data were analyzed using IBM® SPSS® Statistics software (version 27, IBM: NYSE, Armonk, NY, USA). All inferences were based on two-tailed tests, adopting a significance level of 95% (type I error, ɑ=0.05) and a test power of 80% (type II error, β=0.2). Differences in proportions of nominal variables were assessed using the chi-squared test, adjusted by the Bonferroni criteria for multiple comparisons. Numerical and ordinal variables were compared using the Mann-Whitney U-test.

## Results

The sample consisted of 90 volunteers, including 60 participants with highly consistent ADHD symptoms (G1) and 30 participants without symptoms highly consistent with ADHD (G2). No significant differences in age, marital status, or educational level were observed between groups (p>0.05). However, there was a higher prevalence of women (p=0.006), South Americans (p=0.008), and individuals with low/lower middle income (p=0.0001) in G1. The sociodemographic characteristics of the 90 participants are summarized in Table 1.

**Table 1:** Comparisons of demographic variables between subjects with highly consistent ADHD symptoms (G1) and controls (G2) (Median (min-max) and n (%)).

	Groups		
	G1	G2	*p*
	(n=60)	(n=30)	
Age	34.0 (18-57)	32.0 (18-55)	0.46
**Gender**			
Women	55 (91.7)	9 (30)	0.006*
Men	5 (8.3)	21 (70)	
**Race**			
African	1 (1.7)	--	
European	7 (11.6)	14 (46.7)	
Southamerican	46 (76.6)	13 (43.3)	
Arabic	1 (1.7)	2 (6.7)	0.008*
Asian	1 (1.7)	--	
Other	4 (6.7)	1 (3.3)	
**Marital Status**			
Single	26 (43.3)	13 (43.3)	
Divorced	5 (8.3)	2 (6.7)	0.67
Facto Union	7 (11.7)	4 (13.3)	
Married	22 (36.7)	11 (36.7)	
**Education level**			
Primary	2 (3.3)	1 (3.3)	
Secondary	4 (6.7)	--	0.12
Graduate	16 (26.7)	13 (43.4)	
Undergraduate	38 (63.3)	16 (53.3)	
**Income**			
Low / Lower Middle	49 (81.7)	11 (36.7)	
Middle	3 (5)	4 (13.3)	0.0001*
Upper middle	2 (3.3	3 (10)	
High	5 (8.3)	12 (40)	

*p<0.05

Regarding pain-related variables, G1 presented a higher point prevalence of painful TMD (p=0.0001) and headaches (p=0.0001), as well as significantly higher CPI values (p=0.0001). Additionally, G1 showed significantly greater OBC total scores (p=0.001) with higher frequencies in the severe sub-category. Similarly, significantly higher frequencies of self-reported bruxism (p=0.0001) and AB (p=0.003) were found in this group. Importantly, 35 (58.3%) and 25 (41.7%) individuals in G1 reported taking or not taking medication for ADHD treatment, respectively. Table 2 shows comparisons of the assessed clinical variables.

**Table 2:** Findings of clinical variables for highly consistent ADHD symptoms (G1) and controls (G2). Frequencies (n and %) are presented for nominal variables, and means with confidence intervals are provided for numerical variables.

	Groups		
	G1	G2	*p*
	(n=60)	(n=30)	
**Painful TMD**			
Yes	54 (90)	14 (46.7)	0.0001*
No	6 (10)	16 (53.3)	
**Headache**			
Yes	50 (83.3)	14 (46.7)	0.0001*
No	10 (16.7)	16 (53.3)	
**Self-reported Bruxism**			
Yes	52 (86.7)	15 (50)	0.001*
No	8 (13.3)	15 (50)	
**Self-reported SB**			
Yes	19 (31.7)	6 (20)	0.28
No	41 (68.3)	24 (80.0)	
**Self-reported AB**			
Yes	49 (81.7)	15 (50.0)	0.003*
No	11 (18.3)	15 (50.0)	
**Stimulant medications**			
Yes	21 (35.0)	-	----
No	14 (23.3)	-	
**Antidepressant medications**			
Yes	14 (23.3)	-	----
No	21 (35.0)	-	
**CPI**	35.3 (28.5-42.2)	9.8 (2.9-16.6)	0.0001*
**Oral Behaviors**			
OBC total	31.2 (28.2-34.1)	19.9 (15.0-24.8)	0.001*
Normal	3 (5)	12 (40)	
Moderate	13 (21.6)	6 (20)	
Severe	44 (73.3)	12 (40)	

TMD: Temporomandibular Disorders; SB: sleep bruxism; AB: awake bruxism; CPI: Characteristic Pain Intensity; OBC: Oral Behavior Checklist.

Regarding psychosocial status, G1 displayed higher values across all assessed variables: PSS (p=0.0001), PVAQ (p=0.01), PSQI (p=0.0001), JFLS-8 (p=0.007), GAD-7 (p=0.0001), and PHQ-15 (p=0.001), compared to G2. In addition, G1 exhibited higher severe frequencies of perceived stress (PSS), anxiety (GAD-7), somatic symptoms (PHQ-15), and poor sleep quality (PSQI) (Table 3).

**Table 3:** Findings of psychosocial variables for highly consistent ADHD symptoms (G1) and controls (G2). Frequencies (n and %) are presented for nominal variables, and means with confidence intervals are provided for numerical variables.

	Groups		
	G1	G2	*p*
	(n=60)	(n=30)	
**Psychosocial Variables**			
**PSS**	36.6 (34.7-38.6)	24.1 (20.5-27.8)	0.0001*
Low	0 (0)	6 (20)	
Moderate	4 (6.6)	10 (33.3)	
High	56 (93.3)	14 (46.6)	
**PVAQ**	37.1 (33.3-41.0)	29.5 (25.1-33.8)	0.01*
**PSQI**	12.5 (11.4-13.6)	8.3 (6.9-9.7)	0.0001*
Well sleep	3 (5)	7 (23.3)	
Poor sleep	57 (95)	23 (76.6)	
**JFLS-8**	10.4 (7.5-13.4)	4.6 (2.0-7.3)	0.007*
**GAD-7**	13.8 (12.4-15.2)	5.9 (3.7-8.1)	0.0001*
Normal	2 (3.3)	14 (46.6)	
Mild	12 (20)	9 (30)	
Moderate	15 (25)	5 (16.6)	
Severe	31 (51.6)	2 (6.6)	
**PHQ-15**	13.5 (12.1-14.9)	7.2 (5.2-9.2)	0.0001*
Minimal	2 (3.3)	11 (36.6)	
Mild	14 (23.3)	9 (30)	
Moderate	19 (31.6)	7 (23.3)	
Severe	25 (41.6)	3 (10)	

PSS: Perceive Stress Scale; Pain Vigilance and Awareness Questionnaire; PSQI: Pittsburgh Sleep Quality Index; JFLS-8: Jaw Functional Limitation Scale - 8; GAD-7: General Anxiety Disorder – 7; PHQ – 15: Patient Health Questionnaire-15 (somatic symptoms).

## Discussion

The results of this study indicate that individuals in the highly consistent ADHD symptom group reported a greater point prevalence of painful TMD, self-reported bruxism, and awake bruxism, as well as increased levels of psychosocial impairment, compared to control participants. These findings, together with the broader literature on ADHD and pain, suggest that ADHD-related characteristics may be implicated in, or contribute to, the potentiation, predisposition, or persistence of TMD.

The elevated point prevalence of painful TMD among individuals with ADHD symptoms corroborates findings from a previous study,^7^ which identified a robust association between ADHD and musculoskeletal pain conditions, including TMD. This overlap may be partially explained by shared neurobiological mechanisms, particularly dysfunction of dopaminergic pathways involved in both attentional regulation and central pain processing.^18^ This dysregulation may enhance pain sensitivity and compromise endogenous pain modulation, increasing vulnerability to chronic orofacial pain. Additionally, behavioral characteristics commonly associated with ADHD—such as hyperactivity, restlessness, and impulsivity—may promote sustained muscle activity and functional overload of the temporomandibular joints. This hypothesis is supported by findings from a recent study,^17^ in which individuals with ADHD displayed higher rates of parafunctional habits and masticatory muscle pain, suggesting that motor agitation in ADHD may directly contribute to orofacial muscle tension and joint stress. As recommended by the INfORM/IADR key points for good clinical practice, future studies should include validated history taking and clinical assessment by trained clinicians to confirm our TMD findings and support patient-centered decision-making treatment plan.^33^

Our findings demonstrate that self-reported bruxism, particularly AB, was more frequently reported among participants with highly consistent ADHD symptoms. This pattern is consistent with a systematic review and meta-analysis,^34^ which included data from 32 studies and found that children and adolescents with ADHD are nearly eleven times more likely to exhibit AB and almost three times more likely to present with SB compared to their peers without ADHD. Although our study did not identify significant differences in SB between groups, the higher prevalence of AB in the ADHD group may reflect a combination of behavioral, emotional, and pharmacological influences. These findings align with the broader literature indicating that stress, anxiety, and heightened alertness are more prevalent in individuals with ADHD and are also considered primary contributors to AB. In addition, AB may be influenced by psychological factors, particularly emotional reactivity and hypervigilance, and tends to manifest during wakefulness as repetitive clenching, tooth contact, or muscle contraction without tooth contact.^35^ At the same time, the role of medication should not be overlooked. In our sample, several individuals reporting bruxism were also using psychostimulants and/or antidepressants; although we did not analyze these effects in detail, 58.3% of G1 participants reported such medication use. Previous studies have shown that drugs such as methylphenidate and selective serotonin reuptake inhibitors (SSRIs) may trigger or exacerbate bruxism through central mechanisms.^36,37^ Taken together, these observations suggest that, in individuals with ADHD, AB may arise from interacting behavioral, emotional, and pharmacological factors, underscoring the need for careful interpretation and clinical attention to potential orofacial side effects.

In this study, individuals with highly consistent ADHD symptoms reported higher levels of anxiety, somatization, perceived stress, poor sleep quality, and pain hypervigilance—variables commonly associated with chronic pain disorders, including TMD, which was also more prevalent in G1. In addition, most individuals in the highly consistent ADHD symptom group presented higher frequencies in the severe categories across all assessed psychosocial scales. These data are consistent with a recent investigation showing that catastrophizing and hypervigilance significantly influence sleep quality in patients with painful TMD, suggesting a bidirectional relationship between psychological distress and orofacial pain.^38^ Moreover, pain-related disability has been shown to be strongly associated with depression and somatization levels, as well as with TMD pain duration.^6^The elevated scores for pain vigilance and somatic symptoms among highly consistent ADHD symptom participants with painful TMD support the notion that these factors may act as maintaining mechanisms in pain chronification. Additionally, a previous questionnaire-based report showed that the persistence of ADHD symptoms into adulthood was associated with increased somatic stress symptoms and sleep disorders—both known to augment pain sensitivity and bruxism activity—highlighting the interconnected roles of emotional dysregulation and sleep disturbance in exacerbating chronic orofacial pain.^7^

The observed overlap among ADHD symptoms, painful TMD, and self-reported AB underscores the need for interdisciplinary evaluation. Dentists, physicians, and mental health professionals should consider underlying attentional and emotional factors when treating adults with ADHD who present with orofacial pain. Behavioral strategies aimed at reducing oral behaviors that may impact the masticatory system and improving stress regulation may be particularly beneficial for ADHD patients with painful orofacial symptoms. Given the complexity of these interactions, future studies should incorporate objective assessment tools—such as electromyography or polysomnography—along with validated scales, proper clinical diagnoses, and longitudinal follow-up to elucidate causality, temporal dynamics, and the potential impact of pharmacological treatment.

As with any scientific investigation, these findings should be interpreted cautiously within the context of the study’s limitations. In our study, no formal clinical diagnosis of ADHD was performed. Identification of ADHD relied on a validated screening instrument for symptoms highly consistent with ADHD. Additionally, the self-reported data, although commonly used, may be subject to recall bias or subjective misinterpretation. The impact of medications could not be thoroughly assessed, as type, dosage, and duration were not systematically recorded. Moreover, the cross-sectional design performed in this study limits the ability to conclude causal relationships. As an exploratory study, the results should be considered preliminary and hypothesis-generating, providing a rationale for future adequately powered and matched studies to confirm the observed associations. Consequently, the substantial difference between group sizes should be considered as a main limitation since the recommended group ratios for this kind of study design were not reached (ratios up to 1:2). Lastly, it is important to highlight that the use of validated assessment instruments for bruxism, such as the Standardized Tool for the Assessment of Bruxism (STAB), is strongly recommended, as it substantially enhances the methodological rigor and interpretability of the findings of bruxism studies.^39^ By adopting this tool, future studies would improve diagnostic precision, reduce misclassification bias, and facilitate comparability with international research. Therefore, studies employing clinical diagnostic criteria for ADHD, TMD, and bruxism, objective measurement tools, and longitudinal designs will be essential to better evaluate prevalence and associations of these variables and to strengthen causal inferences. This study should thus be regarded as a starting point for future research assessing TMD and bruxism in ADHD populations.

## Conclusion

Adults with symptoms highly consistent with ADHD reported a higher point prevalence of painful TMD, self-reported awake bruxism, and psychosocial impairment compared to individuals without such symptoms.
